# New Diterpenes from Cultures of the Fungus *Engleromyces goetzii* and Their CETP Inhibitory Activity

**DOI:** 10.1007/s13659-015-0055-5

**Published:** 2015-04-08

**Authors:** Yang Wang, Ling Zhang, Fang Wang, Zheng-Hui Li, Ze-Jun Dong, Ji-Kai Liu

**Affiliations:** State Key Laboratory of Phytochemistry and Plant Resources in West China, Kunming Institute of Botany, Chinese Academy of Sciences, Kunming, 650201 China; University of Chinese Academy of Sciences, Beijing, 100049 China

**Keywords:** *Engleromyces goetzii*, Engleromycenolic acid A, Engleromycenolic acid B, Engleromycenol, CETP

## Abstract

**Abstract:**

One new cleistanthane-type diterpene named engleromycenolic acid A (**1**), one new rosane-type diterpene named engleromycenolic acid B (**2**) and one new natural rosane-type diterpene, engleromycenol (**3**), along with three known rosane-type diterpenes, rosololactone (**4**), rosenonolactone (**5**) and 7-deoxyrosenonolactone (**6**) were isolated from cultures of the fungus *Engleromyces goetzii*, where it naturally grows on Alpine bamboo culms. The new compounds were elucidated based on their spectroscopic data. In addition, compounds **1**–**6** were evaluated for their cholesterol ester transfer protein (CETP) inhibition activity. This paper reports the isolation, structural elucidation, and CETP inhibition activity of these compounds.

**Graphical Abstract:**

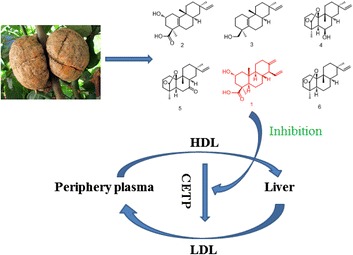

**Electronic supplementary material:**

The online version of this article (doi:10.1007/s13659-015-0055-5) contains supplementary material, which is available to authorized users.

## Introduction

The fungus *Engleromyces goetzii* is widely distributed in Tibet, Sichuan and Yunnan province. It grows on Alpine bamboo culms, and ripens during rainy season from July to August. Local residents usually boil the fruiting bodies in water to treat infection, inflammation and cancer [1–3]. Previous investigation on the fruiting bodies of *E. goetzii* has led to the isolation of neoengleromycin, cytochalasin D and 19,20-epoxycytochalasin D [4–6]. There are no reports about the chemical constituents of the cultures of this fungus.

**Fig. 1:**
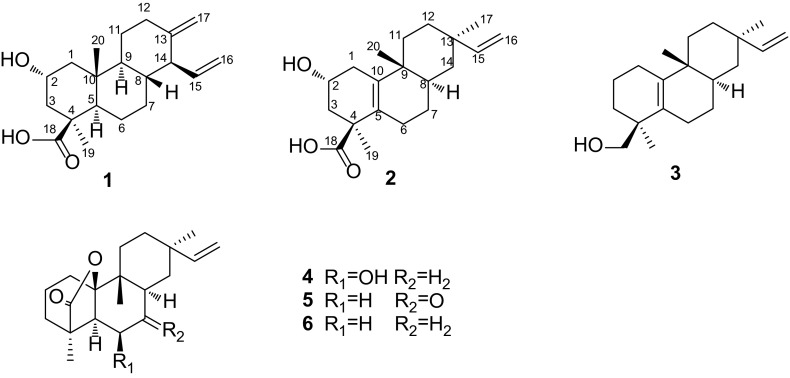
Structures of compounds **1**–**6**

In order to search for more novel and potentially bioactive secondary metabolites, the chemical constituents of *E. goetzii* cultures were investigated by altering the culture conditions of the fungus and enlarging the fermentation scale. This investigation led to the isolation and identification of one new cleistanthane-type diterpene named engleromycenolic acid A (**1**), one new rosane-type diterpene named engleromycenolic acid B (**2**) and one new natural rosane-type diterpene named engleromycenol (**3**), along with three known rosane-type diterpenes: rosololactone (**4**), rosenonolactone (**5**) and 7-deoxyrosenonolactone (**6**) [7]. Their structures (Fig. 1) were elucidated based on the spectroscopic data analyses. The cholesterol ester transfer protein (CETP) inhibition activities of compounds **1**–**6** were examined and engleromycenolic acid A (**1**) showed CETP inhibition activity with IC_50_ value at 7.55 µM.

There are two kinds of lipoproteinsin plasma: one is low-density lipoprotein (LDL) and another is high-density lipoprotein (HDL) [8, 9]. They are in charge of carrying cholesteryl esters in plasma. HDL is responsible for transferring cholesteryl esters from periphery plasma to the liver to metabolize, and LDL mediates the transport of cholesteryl esters from the liver to periphery plasma [8, 10, 11]. CETP promotes the transfer of cholesteryl esters from HDL to LDL. Inhibiting the activity of CETP can promotes the metabolism of cholesteryl esters in the liver and avoids accumulation of cholesterol esters in plasma that may cause atherosclerosis [9, 11–14].

## Results and Discussion

**Fig. 2:**
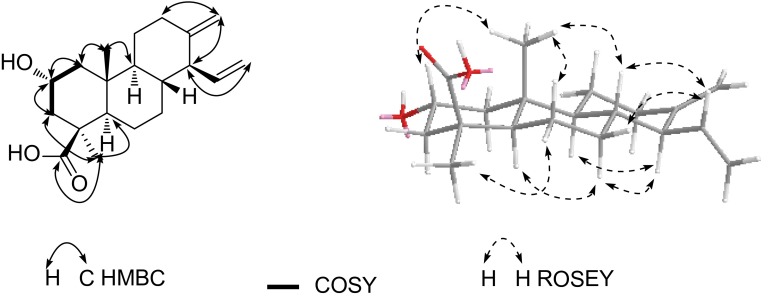
Selected 2D NMR correlations of **1**

Compound **1** was obtained as colorless oil, displayed an [M+Na]^+^ ion at *m/z* 341.2096 on the positive HRESIMS analysis, corresponding to the molecular formula C_20_H_30_O_3_ with six degrees of unsaturation. The ^1^H NMR spectrum of **1** (Table 1) exhibited two methyls at *δ*_H_ 1.28 (3H, s) and 0.80 (3H, s), five olefinic protons at *δ*_H_ 5.69 (1H, ddd, *J* = 17.1, 10.0, 10.0 Hz), 5.16 (1H, dd, *J* = 10.0, 2.2 Hz), 5.00 (1H, dd, *J* = 17.1, 2.2 Hz), 4.67 (1H, br.s) and 4.57 (1H, br.s). The ^13^C NMR spectrum showed 20 carbon resonances, including one carboxyl, four *sp*^2^ olefinic carbons, one oxygen-bearing methine, two tertiary methyls, six methylenes, four methines as well as two quaternary carbons. These data suggested compound **1** might be a tricyclic diterpene. The ^1^H and ^13^C NMR spectral data of **1** were similar to those of auricularic acid [15], which indicated that **1** was a cleistanthane-type diterpene. However, there are certain differences as follows: the resonance of C-2 at *δ*_C_ 19.5 in auricularic acid is down shifted to *δ*_C_ 65.3 in **1**, suggesting that the methylene at C-2 was replaced by an oxygen-bearing methine. Furthermore, the HMBC correlations from *δ*_H_ 2.41, 2.13, 1.00, 0.93 to carbon at *δ*_C_ 65.3, and the ^1^H–^1^H COSY correlations from *δ*_H_ 4.14 to *δ*_H_ 2.41, 2.13, 1.00 and 0.93 in **1**, indicating that the oxygen-bearing methane was placed at C-2. According to the molecular formula, we can conclude the oxygen-bearing methine at C-2 in **1** was substituted by a hydroxyl. In the ROSEY spectrum, the observed cross peaks of CH_3_-20/H-6*β,* H-2/CH_3_-20, CH_3_-20/H-8, H-8/H-15 and H-15/H-7*β* suggested that H-2, H-8, H-15 H-7*β*, H-6*β* and CH_3_-20 were in the same side, whereas the cross peaks of H-6*α*/CH_3_-19, H-9/H-14, H-7*α*/H-14 and H-7*α*/H-5 indicated that H-6*α*, CH_3_-19, H-9, H-14, H-7*α*, H-14 and H-5 lied on the opposite side (Fig. 2). Therefore, **1** was determined as engleromycenolic acid A.

**Table 1:** ^1^H and ^13^C NMR spectroscopic data for compound **1**

Pos.	*δ*_C_ Type	*δ*_H_ (*J* in Hz)
1	49.3, t	2.13 (ddd 12.3, 4.3, 1.9, H-*β*) 0.93, overlapped
2	65.3, d	4.14 (tt, 11.5,4.3, H-*β*)
3	47.7, t	2.41 (ddd 12.3, 4.3, 1.9, H-*β*) 1.00, overlapped
4	46.0, s	
5	56.6, d	1.15, overlapped
6	24.3, t	1.92, overlapped 1.74 (dddd 13.5, 13.5, 13.5, 3.5, H-*β*)
7	35.3, t	2.02 (dq-like, 13.3, 3.6, H-*β*) 0.89 (qd-like, 13.3, 3.6, H-*α*)
8	42.8, d	1.17, overlapped
9	55.8, d	1.03, overlapped
10	40.0, s	
11	28.1, t	1.93, overlapped, H-*α* 1.12, overlapped, H-*β*
12	37.0, t	2.45 (ddd, 13.1, 3.2, 3.2, H-*β*) 2.06, overlapped
13	152.4, s	
14	56.0, d	2.28 (dd, 10.0, 10.0)
15	141.2, d	5.69 (ddd, 17.1, 10.0, 10.0)
16	116.9, t	5.16 (dd,10.0, 2.2) 5.00 (dd, 17.1, 2.2)
17	106.8, t	4.67 br.s 4.57 br.s
18	181.2, s	
19	29.5, q	1.28, s
20	14.4, q	0.80, s

Spectra were measured in CD_3_OD at 600 MHz

**Fig. 3:**
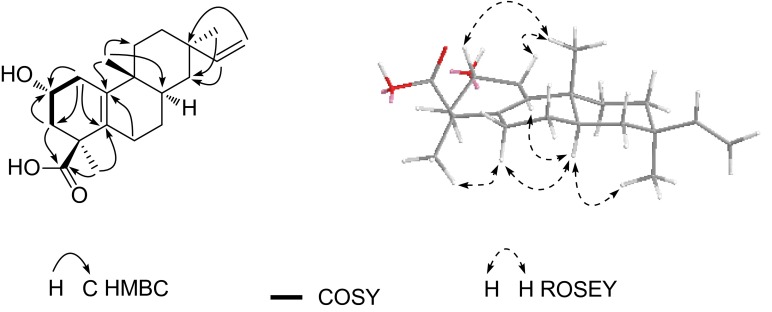
Selected 2D NMR correlations of **2**

Compound **2** was isolated as white powder. The molecular formula C_20_H_30_O_3_ was determined by the HRESIMS analysis (*m/z* 341.2091 [M+Na]^+^), which indicated six degrees of unsaturation. The ^1^H NMR spectrum (Table 2) of **2** exhibited three olefinic protons at *δ*_H_ 5.87 (1H, dd, *J* = 17.5, 10.7 Hz), 4.97 (1H, dd, *J* = 17.5, 1.0 Hz) and 4.88 (1H, dd, *J* = 10.7, 1.0 Hz), one oxygen-bearing methine at *δ*_H_ 3.95 (1H, m) and three methyls at *δ*_H_ 0.98 (3H, s), 1.08 (3H, s) and 1.31 (3H, s). The ^13^C NMR spectrum showed 20 carbon resonances including one carbonyl, four olefinic carbons, one oxygen-bearing methine, three methyls, seven methylenes, one methine and three quaternary carbons. Comparison of NMR data of **2** with those of 7-deoxyrosenonolactone (**6**) [16], revealed the presence of the characteristic signals of a rosane-type diterpene. The resonances at *δ*_C_ 129.5 and 138.8 in the ^13^C NMR spectrum of **2** suggested there was an additional double bond in **2**. The HMBC correlations from *δ*_H_ 2.42 and 1.86 to *δ*_C_ 129.5, *δ*_H_ 1.31 to *δ*_C_ 129.5; *δ*_H_ 2.22 and 1.97 to *δ*_C_ 138.8 as well as *δ*_H_ 0.98 to *δ*_C_ 138.8, indicated that the new double bond located between C-5 and C-10. Based on the analysis, the oxygen linkage between C-10 and C-18 in **6** was broken and formed a carboxyl at C-18 in **2**, which was confirmed by the HMBC correlations from *δ*_H_ 2.21, 1.41 and 1.31 to *δ*_C_ 180.9. In addition, the resonance of *δ*_C_ 21.7 (t) in **6** is down shifted to *δ*_C_ 66.2 (d) in **2**, indicated the methylene is oxidized by a hydroxyl. The HMBC correlations from *δ*_H_ 2.42, 2.21, 1.86 and 1.41 to carbon at *δ*_C_ 66.2, as well as the ^1^H–^1^H COSY correlations from *δ*_H_ 3.95 to *δ*_H_ 2.42, 2.21, 1.86 and 1.41, suggesting the hydroxyl was attached to C-2. The ROSEY spectrum showed cross peaks between CH_3_-20/H-2, H-1*β*/CH_3_-20, H-1*α*/H-8, H-8/H-6*α*, CH_3_-19/H-6*α* and CH_3_-17/H-8 suggested CH_3_-20, H-2, H-1*β* in the same side, and H-1*α*, H-8, H-6*α*, CH_3_-19 and CH_3_-17 in the opposite side (Fig. 3). The detailed analysis of chemical shift and coupling constant led to the determination that compound **2** was elucidated as engleromycenolic acid B.

**Table 2:** ^1^H and ^13^C NMR spectroscopic data for compounds **2** and **3**

Pos.	2^a^		3^b^	
	*δ*_C_ Type	*δ*_H_ (*J* in Hz)	*δ*_C_ Type	*δ*_H_ (*J* in Hz)
1	35.4, t	2.42 (dd, 15.9, 3.8, H-*β*) 1.86, m, H-*α*	25.0, t	1.98, overlapped
2	66.2, d	3.95, m	19.7, t	1.68, overlapped 1.58, overlapped
3	45.8, t	2.21, overlapped 1.41, overlapped	34.9, t	1.78 (ddd, 13.5, 5.0, 3.5, H-*β*) 1.27, overlapped
4	49.8, s		39.3, s	
5	129.5, s		128.7, s	
6	28.1, t	2.22, overlapped, H-*α* 1.97 (dd,16.9, 5.4, H-*β*)	25.2, t	2.10, overlapped 1.96, overlapped
7	26.5, t	1.51, overlapped 1.34, overlapped	25.8, t	1.39, overlapped 1.32, overlapped
8	38.9, d	1.65, overlapped	37.5, d	1.59, overlapped
9	38.6, s		37.8, s	
10	138.8, s		142.5, s	
11	32.6, t	1.68, overlapped 1.39, overlapped	31.7, t	1.61, overlapped 1.33, overlapped
12	33.5, t	1.64, overlapped 1.33, overlapped	32.7, t	1.54, overlapped 1.27, overlapped
13	37.3, s		36.4, s	
14	40.6, t	1.48, overlapped, H-*β* 1.13 (br.d, 13.1, H-*α*)	39.8, t	1.38, overlapped 1.07, overlapped
15	152.2, d	5.87 (dd,17.5, 10.7)	151.4, d	5.82 (dd, 17.4, 10.7)
16	109.2, t	4.97 (dd, 17.5, 1.0) 4.88 (dd, 10.7, 1.0)	108.8, t	4.92 (dd, 17.4, 1.2) 4.85 (dd, 10.7, 1.2)
17	23.5, q	1.08, s	23.1, q	1.03, s
18	180.9, s		69.9, t	3.58(d, 10.8) 3.32(d, 10.8)
19	25.3, q	1.31, s	23.6, q	0.95, s
20	16.7, q	0.98, s	17.9, q	0.88, s

^a^Spectra were measured in CD_3_OD at 600 MHz

^b^Spectra were measured in CDCl_3_ at 400 MHz

**Fig. 4:**
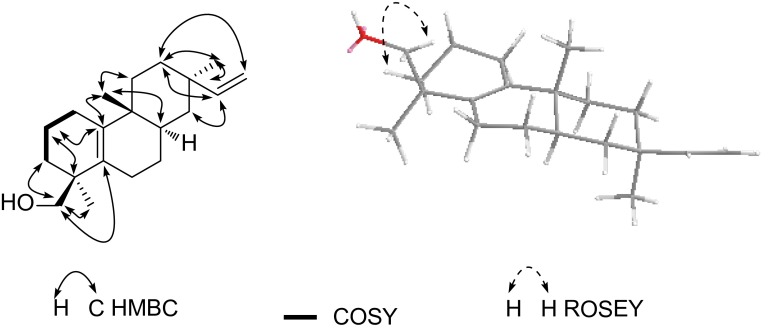
Selected 2D NMR correlations of **3**

Compound **3** possessed a molecular formula C_20_H_32_O as determined by the HRESIMS (*m/z* 311.2354, [M+Na]^+^), which implied five degrees of unsaturation. The 1D NMR spectroscopic data (Table 2) suggested that the backbone of **3** was the same as that of **2**. Differences between them were identified to be the loss of a carbonyl and an oxygen-bearing methine, as well as the appearance of a hydroxymethyl and a methylene. The HMBC correlations of *δ*_H_ 3.58 and 3.32 with *δ*_C_ 23.6, 34.9 and 128.7 suggested the carbonyl at C-18 in **2** is replaced by the hydroxymethyl (*δ*_C_ 69.9, *δ*_H_ 3.58 and 3.32) in **3**. In addition, the HMBC correlation from *δ*_H_ 1.68 and 1.58 to *δ*_C_ 142.5 and 39.3, and the ^1^H–^1^H COSY correlation from *δ*_H_ 1.98 to *δ*_H_ 1.68 and 1.58, *δ*_H_ 1.68 and 1.58 to *δ*_H_ 1.78 and 1.27, indicated that the oxygen-bearing methine at C-2 in **2** was replaced by a methylene in **3**. The configuration of C-8, C-9 and C-13 were established by comparing the NMR data of **3** with **2**. The H-3 signal at *δ*_H_ 1.78 (1H, ddd, *J* = 13.5, 5.0, 3.5 Hz) suggested it to be equatorial *β*-oriented. In the ROSEY spectrum, the observed cross peak of *δ*_H_ 1.78 and 3.32 indicated the hydroxymethyl group in C-4 was located in axial *β*-oriented. In addition, there are no cross peak between H-18 (3.58, 3.32) and H-3*α* (1.27) in ROSEY spectrum, which further approved the conclusion above (Fig. 4). According to this analysis, compound **3** was confirmed as engleromycenol.

Based on the spectroscopic analyses and the comparison with the literature, the known compounds were identified as rosololactone (**4**) [17, 18], rosenonolactone (**5**) [18] and 7-deoxyrosenonolactone (**6**) [16]. Compound **3** has been reported in the conversion of 7-deoxyrosenonolactone to **3** by Connolly [19]. However, there are no reports about the NMR data of **3**.

All the isolates were assayed for their CETP inhibition activity with the CETP Inhibitor Drug Screening Kit. The result showed that compounds **2**–**6** exhibited weak inhibition activity of CETP, however, engleromycenolic acid A (**1)** significantly inhibited the activity of CETP with the IC_50_ value at 7.55 µM. Results of the study suggested that engleromycenolic acid A (**1**) might be a good candidate to develop effective therapeutic agent for the treatment of atherosclerotic cardiovascular diseases.

## Experimental Section

### General Experimental Procedures

The optical rotations were measured on a JASCO model 1020 polarimeter (JASCO International Co., Ltd., Tokyo, Japan). The IR spectra were obtained on a Bruker TENSOR 27FT-IR spectrometer (Bruker, Ettlingen, Germany) using KBr pellets. The 1D and 2D NMR data were performed on Bruker Avance III 600 and AM-400 instruments (Bruker, Rheinstetten, Germany) at room temperature. The chemical shifts (*δ*) were expressed in ppm with reference to the solvent signals. The mass spectra (MS) were acquired on an API QSTAR time-of-flight mass spectrometer (MDS Sciex, Ontario, Canada) or a VG Autospec-3000 spectrometer (VG, Manchester, England). Silica gel (200–300 mesh, Qingdao Marine Chemical Inc., Qingdao, China), Sephadex LH-20 (Amersham Biosciences, Sweden), and RP-18 gel (40–75 µm, Fuji Silysia Chemical Ltd., Japan) were used for column chromatography. Preparative HPLC (Prep-HPLC) was performed on an Agilent 1100 liquid chromatography system equipped with a ZORBAX SB-C_18_ column (9.4 mm × 150 mm). Precoated silica gel GF254 plates (Qingdao Marine Chemical Inc., Qingdao, China) were used for TLC analysis. The fractions were monitored by TLC analysis, and spots were visualized under UV light (254 or 365 nm) or by heating silica gel plates sprayed with 10 % H_2_SO_4_ in ethanol.

### Fungal Material and Cultivation Conditions

Fruiting bodies of *E. goetzii* were collected from Shangri-La county in Yunnan Province, China. A voucher specimen has been deposited in the Herbarium of the Kunming Institute of Botany of the Chinese Academy of Sciences. The mycelia cultures were derived from the tissue plugs. The culture PDA medium consisted of glucose (5 %), peptone from porcine meat (0.15 %), yeast powder (0.5 %), KH_2_PO_4_ (0.05 %) and MgSO_4_ (0.05 %). The inoculums of *E. goetzii* were prepared in a 15 L-fermentation tank for 6 days under the following conditions: culture temperature, 24 °C; initial pH, 6.0; agitation speed, 250 r/min; inoculation volume, 10 % (by volume); and aeration rate, 1.0 volume/culture volume/min. Subsequently, the liquid seed was transferred into a 100 L-fermentation tank for cultivation under the same conditions for 20 days to afford an 80 L culture broth.

### Extraction and Isolation

The fermentation broth (80 L) was filtered, and the filtrate was concentrated to 10 L under reduced pressure and then extracted with ethyl acetate (3 × 10 L). The organic layer was evaporated to give a crude extract (350 g). Subsequently, the extract was subjected to silica gel column chromatography, using a petroleum ether/acetone gradient (100:0 → 0:100 V/V) to afford fractions F_1_–F_7_ based on TLC analysis. F_3_ was purified using Sephadex LH-20 column chromatography (chloroform/methanol = 1:1 V/V) and then subjected to silica gel column chromatography (petroleum ether/acetone = 100:1 V/V) to afford compound **6** (50.0 mg). F_4_ was fractioned by Sephadex LH-20 (chloroform/methanol = 1:1 V/V) and then subjected to silica gel with a petroleum ether/acetone system (150:1 V/V) to give compound **5** (15.1 mg). F_5_ was separated by silica gel column chromatography (chloroform/methanol = 100:1 V/V) to yield F_5-1_ and F_5-2_. F_5-1_ was further purified by Sephadex LH-20 column chromatography (chloroform/methanol = 1:1 V/V) to afford compound **4** (13.3 mg). F_5-2_ was separated with silica gel column chromatography (chloroform/methanol = 150:1 V/V) and Sephadex LH-20 column chromatography (chloroform/methanol = 1:1 V/V) to give F_5-2-1_ and F_5-2-2_. F_5-2-1_ was separated by preparative HPLC (acetonitrile/water = 3:7 → 6:4 V/V) to provide compound **1** (18.0 mg) and **2** (10.6 mg). F_7_ was purified by Sephadex LH-20 column chromatography (chloroform/methanol = 1:1 V/V) and RP-18 gel (methanol/water = 1:1 V/V) to give compound **3** (30.8 mg).

### Engleromycenolic Acid A (**1**)

Colorless oil; $[\alpha ]_{\text{D}}^{21.2}$ +30.5 (*c* 0.23, MeOH); IR (KBr) $v_{max}$ 3446, 3080, 2943, 2863, 1693, 1642, 1467, 1447, 1200 cm^−1^; ^1^H and ^13^C NMR data see Table 1; ESIMS (negative) *m/z* 317 (100) [M−H]^−^; HRESIMS (positive) *m/z* 341.2096 [M+Na]^+^ (calcd for C_20_H_30_O_3_Na, 341.2093).

### Engleromycenolic Acid B (**2**)

White powder; $[\alpha ]_{\text{D}}^{24.6}$ –166.3 (*c* 0.32, MeOH); IR (KBr) $v_{max}$ 3440, 3082, 2928, 2871, 1700, 1635, 1465, 1233 cm^−1^; ^1^H and ^13^C NMR data see Table 2; ESIMS (negative) *m/z* 317 (100) [M−H]^−^; HRESIMS (positive) *m/z* 341.2091 [M+Na]^+^ (calcd for C_20_H_30_O_3_Na, 341.2093).

### Engleromycenol (**3**)

White powder; $[\alpha ]_{\text{D}}^{24.5}$ −139.1 (*c* 0.13, MeOH); IR (KBr) $v_{max}$ 3440, 3082, 2927, 2876, 1637, 1462, 1434, 1376, 1199 cm^−1^; ^1^H and ^13^C NMR data see Table 2; ESIMS (positive) *m/z* 311 (40) [M+Na]^+^; HRESIMS (positive) *m/z* 311.2354 [M+Na]^+^ (calcd for C_20_H_32_ONa, 311.2351).

### CETP Inhibition Activity Assay

Cholesterol ester transfer protein inhibition activity assay were carried out using a CETP Inhibitor Screening Kit (BioVision Incorporated., Milpitas, USA). CETP is a member of the lipid transfer/lipopolysaccharide binding protein gene family. CETP transfers neutral lipids from HDL to LDL and is present in normal human plasma and serum. The CETP Drug Screening Kit uses a donor molecule containing a fluorescent self-quenched neutral lipid that is transferred to an acceptor molecule in the presence of CETP (rabbit serum). CETP-mediated transfer of the fluorescent neutral lipid to the acceptor molecule results in an increase in fluorescence (ExEm = 465/535 nm). Inhibitor of CETP will inhibit the lipid transfer and subsequently decrease fluorescence intensity. The assay was carried out in a microtiter plate. Reagents were kept on ice prior to setting up the assay. The reaction mixture, containing test sample in 160 µL dH_2_O or control vehicle (160 µL dH_2_O); 20 µL CETP assay buffer; 10 µL of donor molecule and 10 µL of acceptor molecule was mixed well. The reaction was initiated by the addition of 3 µL of rabbit serum. After 60 min of incubation at 37 °C, transfer was measured by the fluorescence intensity with BioTek Instrument (Gene Company Limited., USA). Background values were obtained from a blank with 160 µL dH_2_O. Percent inhibition of CETP activity was calculated by subtracting the background values from both control and test sample values. The IC_50_ value was calculated by Reed and Muench’s method.

$$ \% {\text{ Inhibition }} = { 1}00 \, \times \left[ {1 - \frac{{{\rm Sample}\;{\rm (fluorescence}\;\text{intensity}) - {\rm Background}\;{\rm (fluorescence}\;\text{intensity})}}{{{\rm Control}\;{\rm (fluorescence}\;\text{intensity}) - {\rm Background}\;{\rm (fluorescence}\;\text{intensity})}}} \right] $$

## Electronic supplementary material

Supplementary material 1 (PDF 1738 kb)
