# Supplementary material for: New Diterpenes from Cultures of the Fungus Engleromyces goetzii and Their CETP Inhibitory Activity
Source: Nat Prod Bioprospect. 2015 Apr 8;5(2):69–75. doi: 10.1007/s13659-015-0055-5 (PMC4402584; doi:10.1007/s13659-015-0055-5)
Supplement: Supplementary file 1 — Supplementary material 1 (PDF 1738 kb) [file 13659_2015_55_MOESM1_ESM.pdf]

Cleistanthane-type and Rosane-type diterpenes from the cultures of *Engleromyces goetzii* and their CETP inhibition activity

Yang Wang[a,b]*, Ling Zhang*[a]*, Ze-Jun Dong*[a]*, Zheng-Hui Li*[a]*, Ji-Kai Liu*[a,*]

[a]*State Key Laboratory of Phytochemistry and Plant Resources in West China, Kunming Institute of Botany, Chinese Academy of Science, Kunming 650201, China*

[b]*Graduate University of Chinese Academy of Science, Beijing 100039, China*

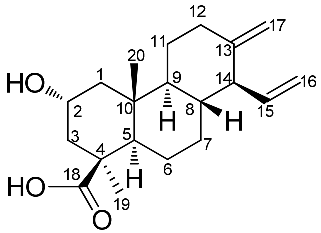

1

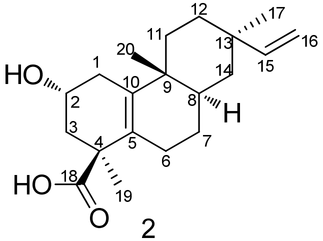

2

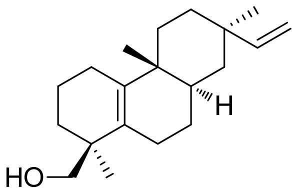

3

Contents:

S1. ^{1}H NMR spectrum (600 MHz, CD_3OD) of 2α-hydroxyl auricularic acid (**1**)

S2. ^{13}C NMR spectrum (100 MHz, CD_3OD) of 2α-hydroxyl auricularic acid (**1**)

S3. HSQC spectrum (600 MHz, CD_3OD) of 2α-hydroxyl auricularic acid (**1**)

S4. HMBC spectrum (600 MHz, CD_3OD) of 2α-hydroxyl auricularic acid (**1**)

S5. COSY spectrum (600 MHz, CD_3OD) of 2α-hydroxyl auricularic acid (**1**)

S6. ROSEY spectrum (600 MHz, CD_3OD) of 2α-hydroxyl auricularic acid (**1**)

S7. HR-ESI-MS spectrum of 2α-hydroxyl auricularic acid (**1**)

S8. ^{1}H NMR spectrum (600 MHz, CD_3OD) of rosenolic acid (**2**)

S9. ^{13}C NMR spectrum (150 MHz, CD_3OD) of rosenolic acid (**2**)

S10. HSQC spectrum (600 MHz, CD_3OD) of rosenolic acid (**2**)

S11. HMBC spectrum (600 MHz, CD_3OD) of rosenolic acid (**2**)

S12. COSY spectrum (600 MHz, CD_3OD) of rosenolic acid (**2**)

S13. ROSEY spectrum (600 MHz, CD_3OD) of rosenolic acid (**2**)

S14. HR-ESI-MS spectrum of rosenolic acid (**2**)

S15. ^1H NMR spectrum (400 MHz, $CDCl_3$) of rosenolic dienol (**3**)

S16. ^{13}C NMR spectrum (100 MHz, $CDCl_3$) of rosenolic dienol (**3**)

S17. HSQC spectrum (500 MHz, $CDCl_3$) of rosenolic dienol (**3**)

S18. HMBC spectrum (500 MHz, $CDCl_3$) of rosenolic dienol (**3**)

S19. COSY spectrum (500 MHz, $CDCl_3$) of rosenolic dienol (**3**)

S20. ROSEY spectrum (500 MHz, $CDCl_3$) of rosenolic dienol (**3**)

S21. HR-ESI-MS spectrum of rosenolic dienol (**3**)

S1. ^{1}H NMR spectrum (600 MHz, CD_3OD) of 2α-hydroxyl auricularic acid (**1**)

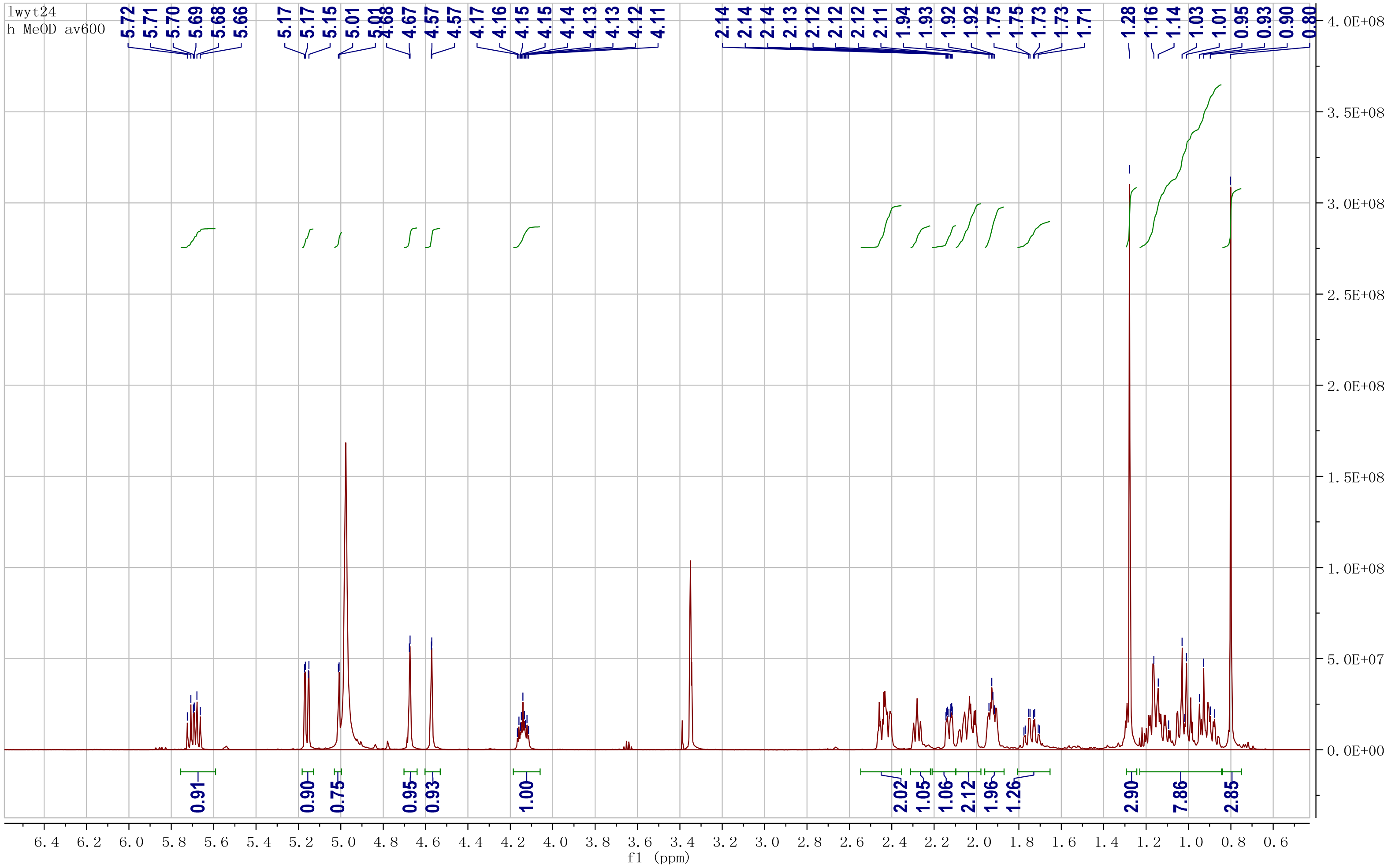

S2. ^{13}C NMR spectrum (100 MHz, CD_3OD) of 2α-hydroxyl auricularic acid (**1**)

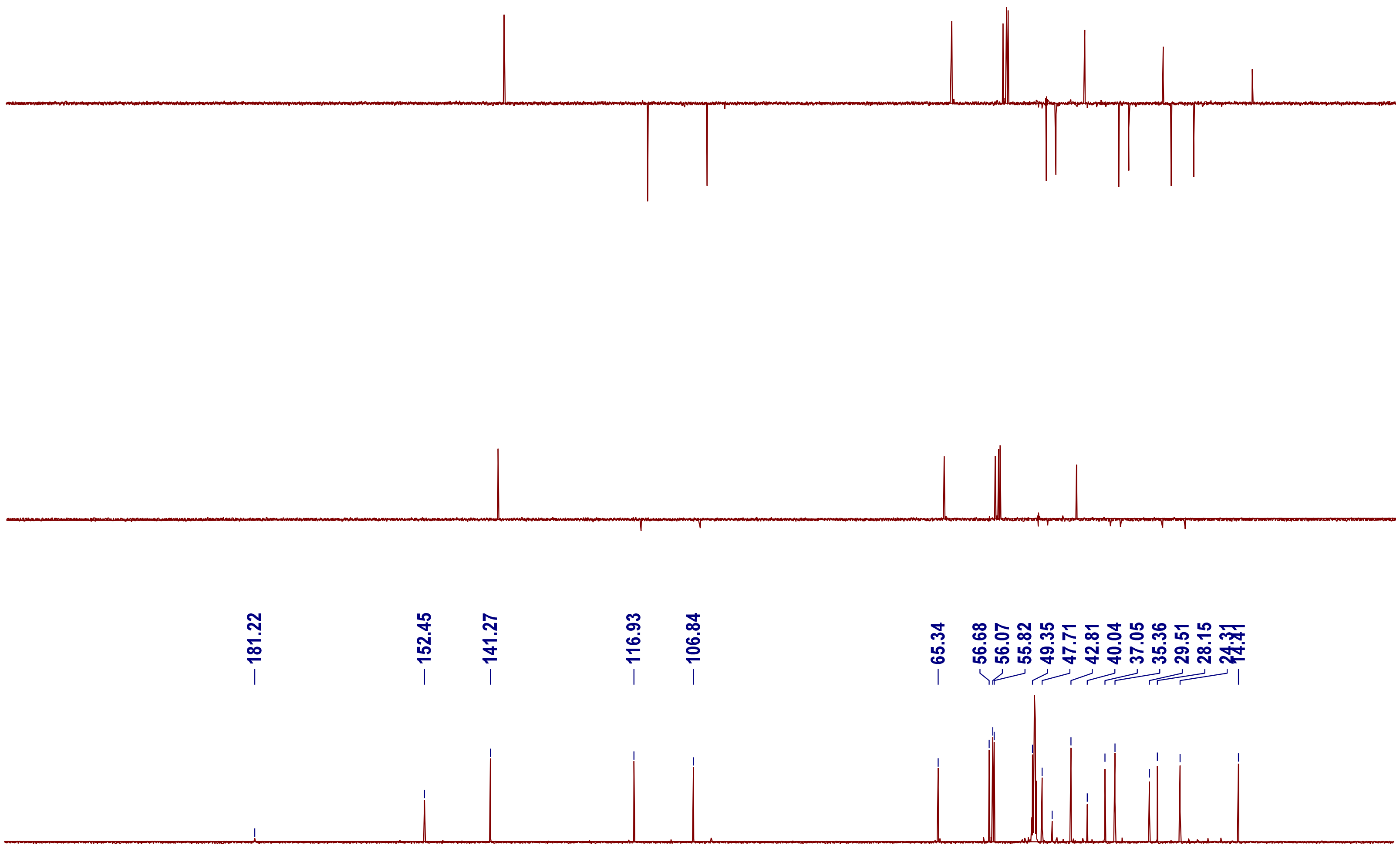

S3. HSQC spectrum (600 MHz, CD_3OD) of 2α-hydroxyl auricularic acid (**1**)

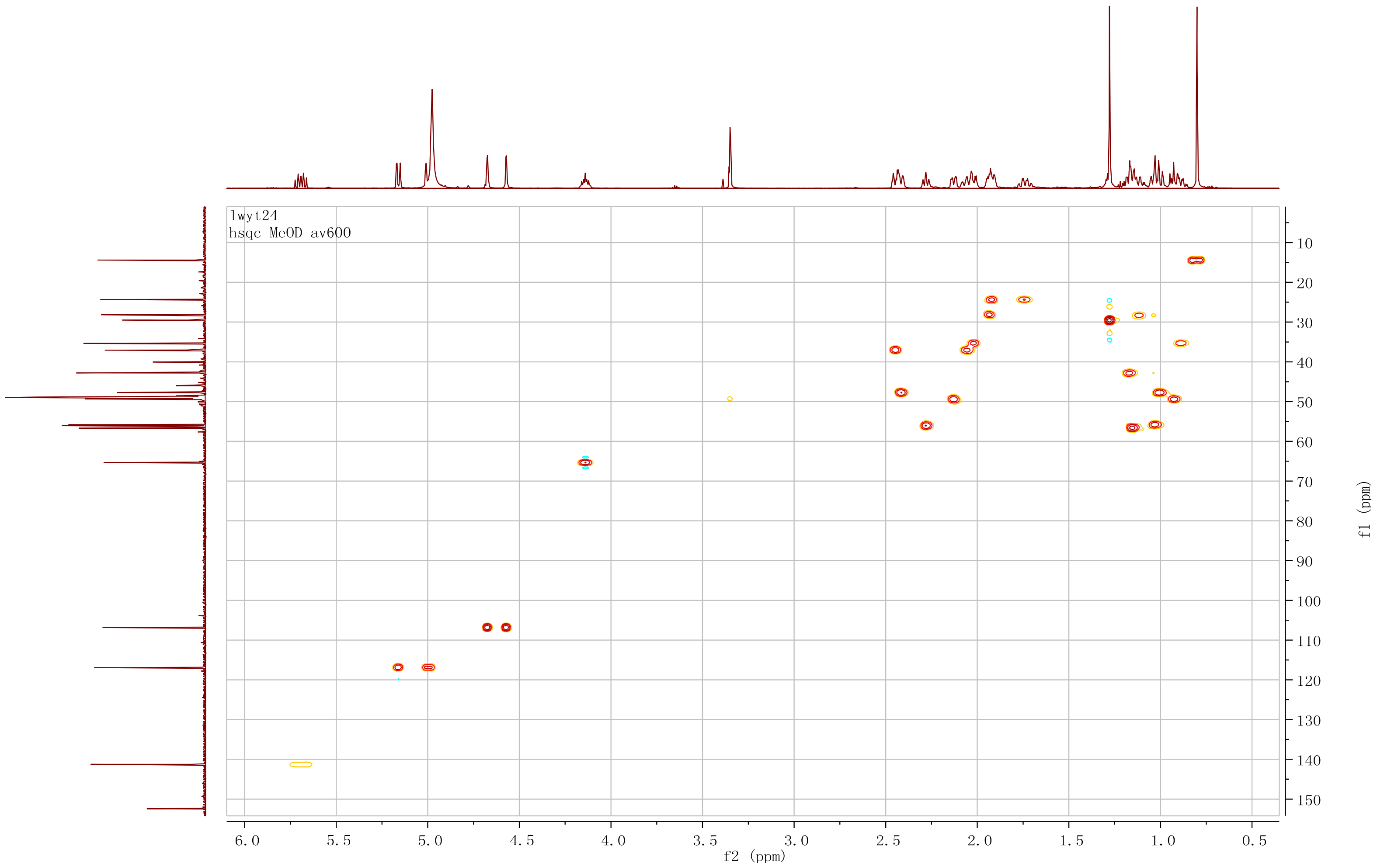

S4. HMBC spectrum (600 MHz, CD_3OD) of 2α-hydroxyl auricularic acid (**1**)

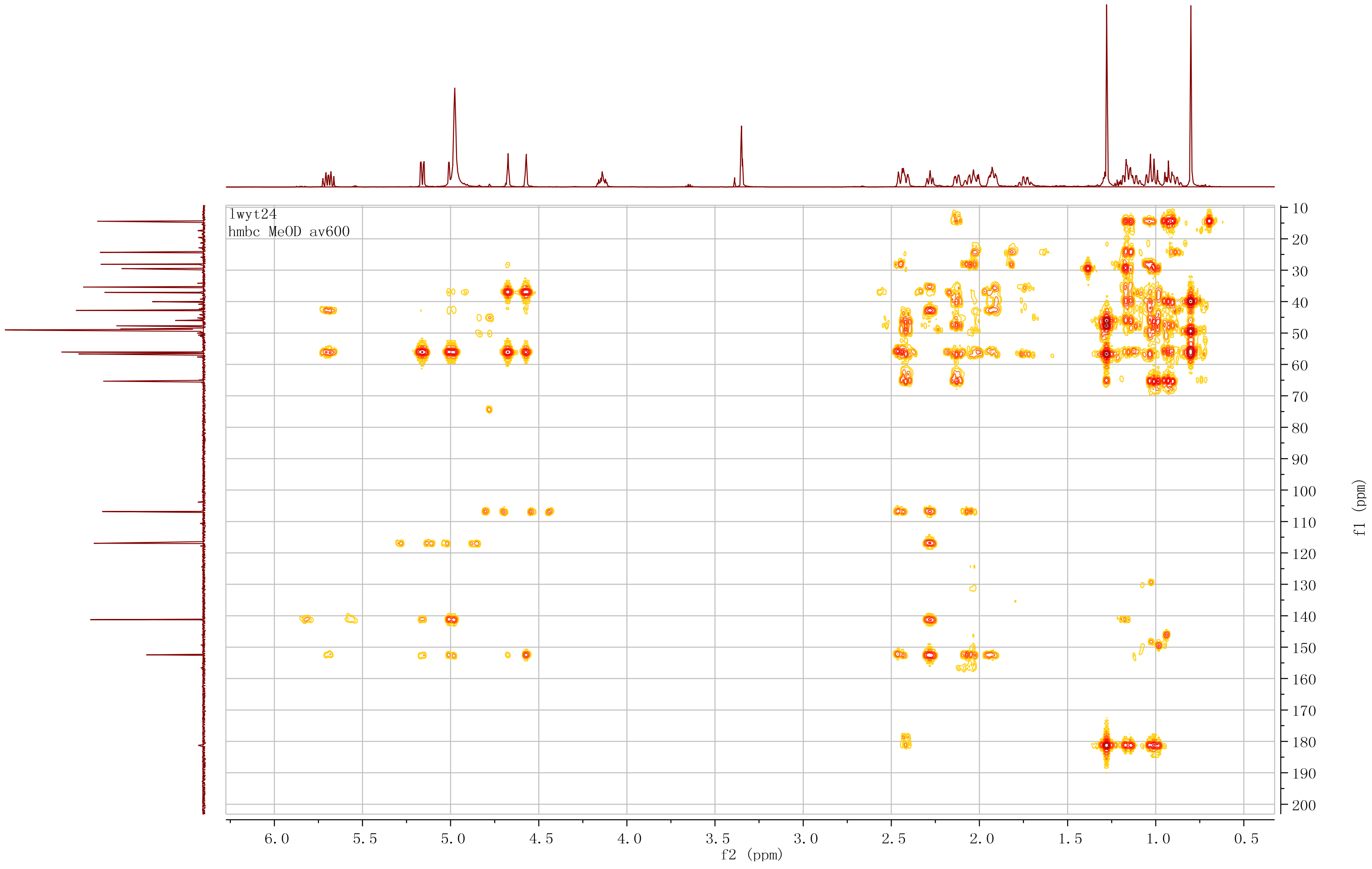

S5. COSY spectrum (600 MHz, CD_3OD) of 2α-hydroxyl auricularic acid (**1**)

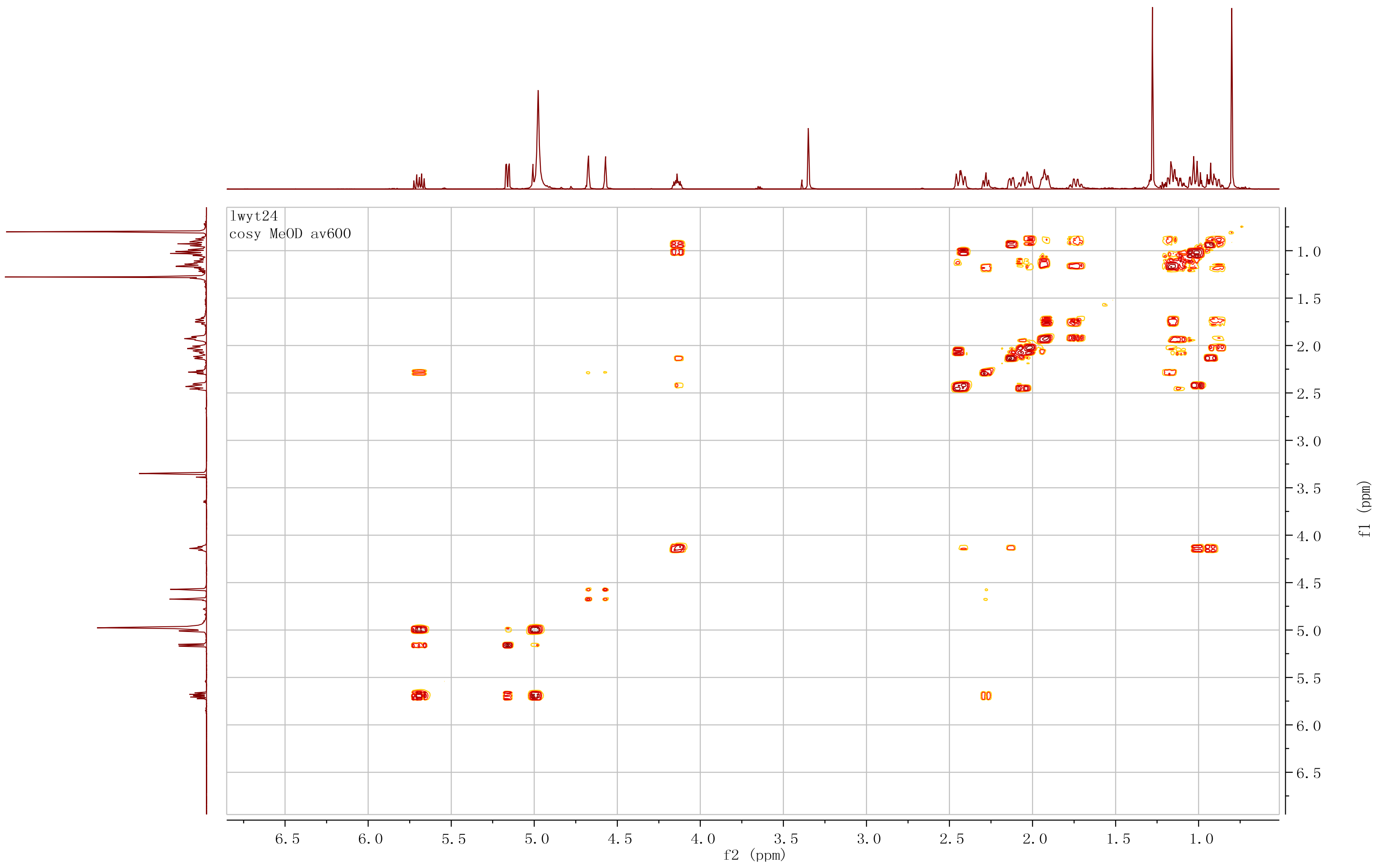

S6. ROSEY spectrum (600 MHz, CD_3OD) of 2α-hydroxyl auricularic acid (**1**)

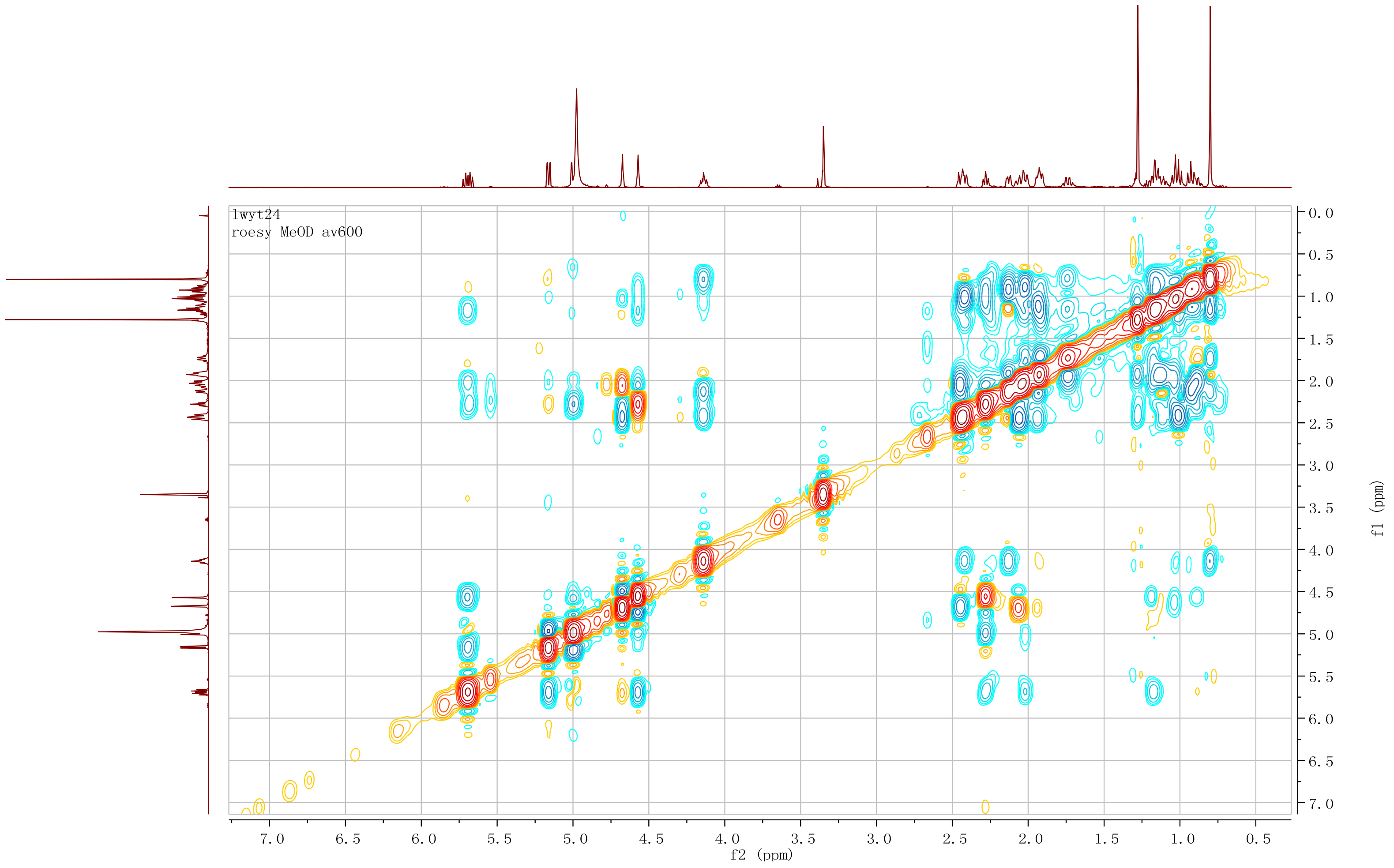

S7. HR-ESI-MS spectrum of 2α-hydroxyl auricularic acid (**1**)

Qualitative Analysis Report

**Data Filename**	1403125ESIA2.d	**Sample Name**	lwyt24
**Sample Type**	Sample	**Position**	
**Instrument Name**	Agilent G6230 TOF MS	**User Name**	KIB
**Acq Method**	ESI.m	**Acquired Time**	3/25/2014 10:16:35 AM
**IRM Calibration Status**	Success	**DA Method**	ESIN.m
**Comment**			

**Sample Group**		**Info.**
**Acquisition SW**	6200 series TOF/6500 series	
**Version**	Q-TOF B.05.01 (B5125.1)	

User Spectra

Fragmentor Voltage	Collision Energy	Ionization Mode
200	0	ESI

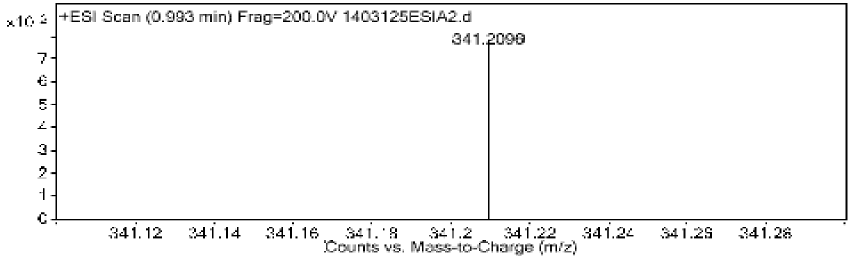

Peak List

m/z	z	Abund
922.0098	1	221952.64

Formula Calculator Element Limits

Element	Min	Max
C	0	200
H	0	400
O	2	4
Na	1	1

Formula Calculator Results

Formula	CalculatedMass	Mz	Diff.(mDa)	Diff. (ppm)	DBE
C20 H30 Na O3	341.2093	341.2096	-0.3	0.9	5.5

--- End Of Report ---

S8. ^{1}H NMR spectrum (600 MHz, CD_3OD) of rosenolic acid (**2**)

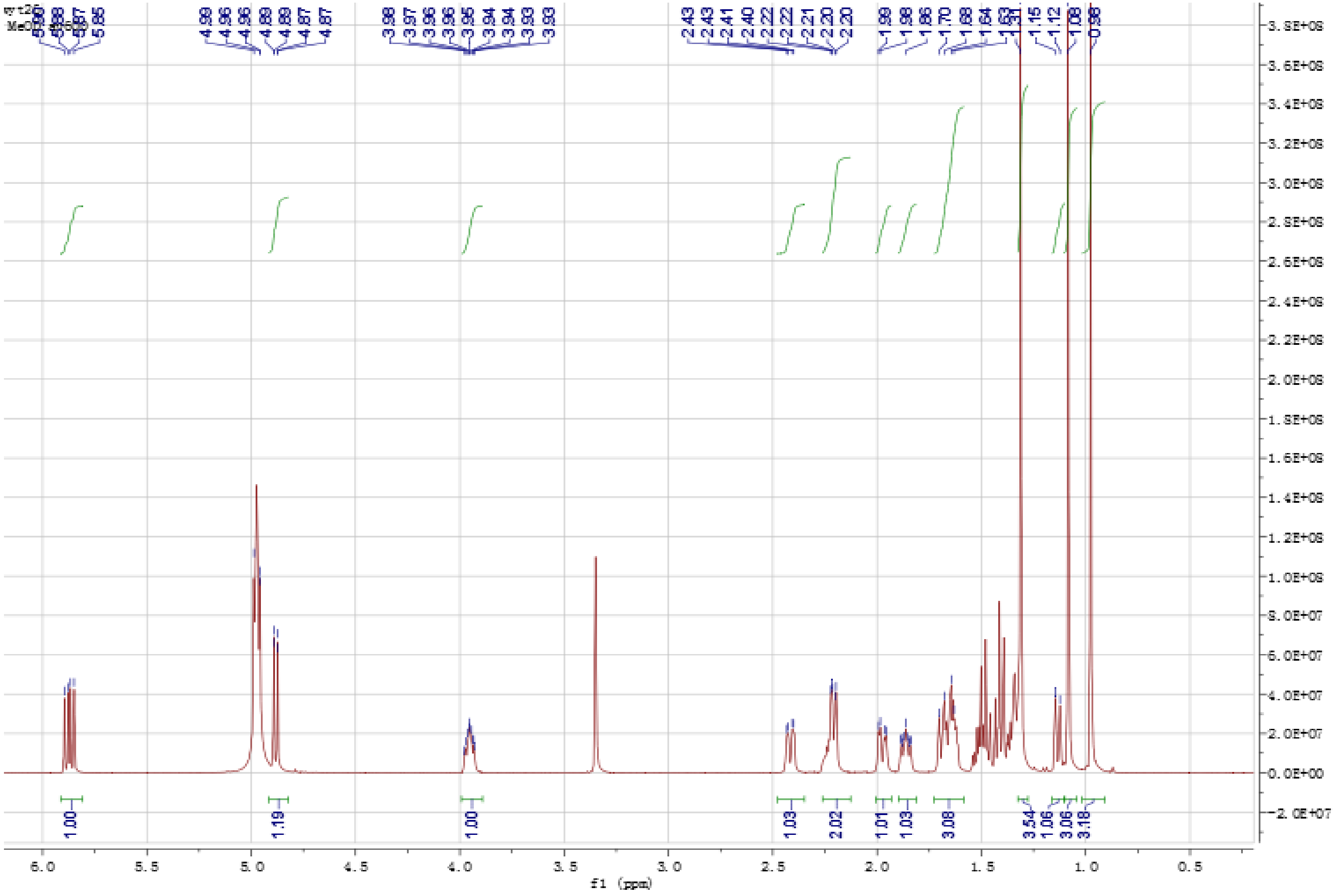

S9. ^{13}C NMR spectrum (150 MHz, CD_3OD) of rosenolic acid (**2**)

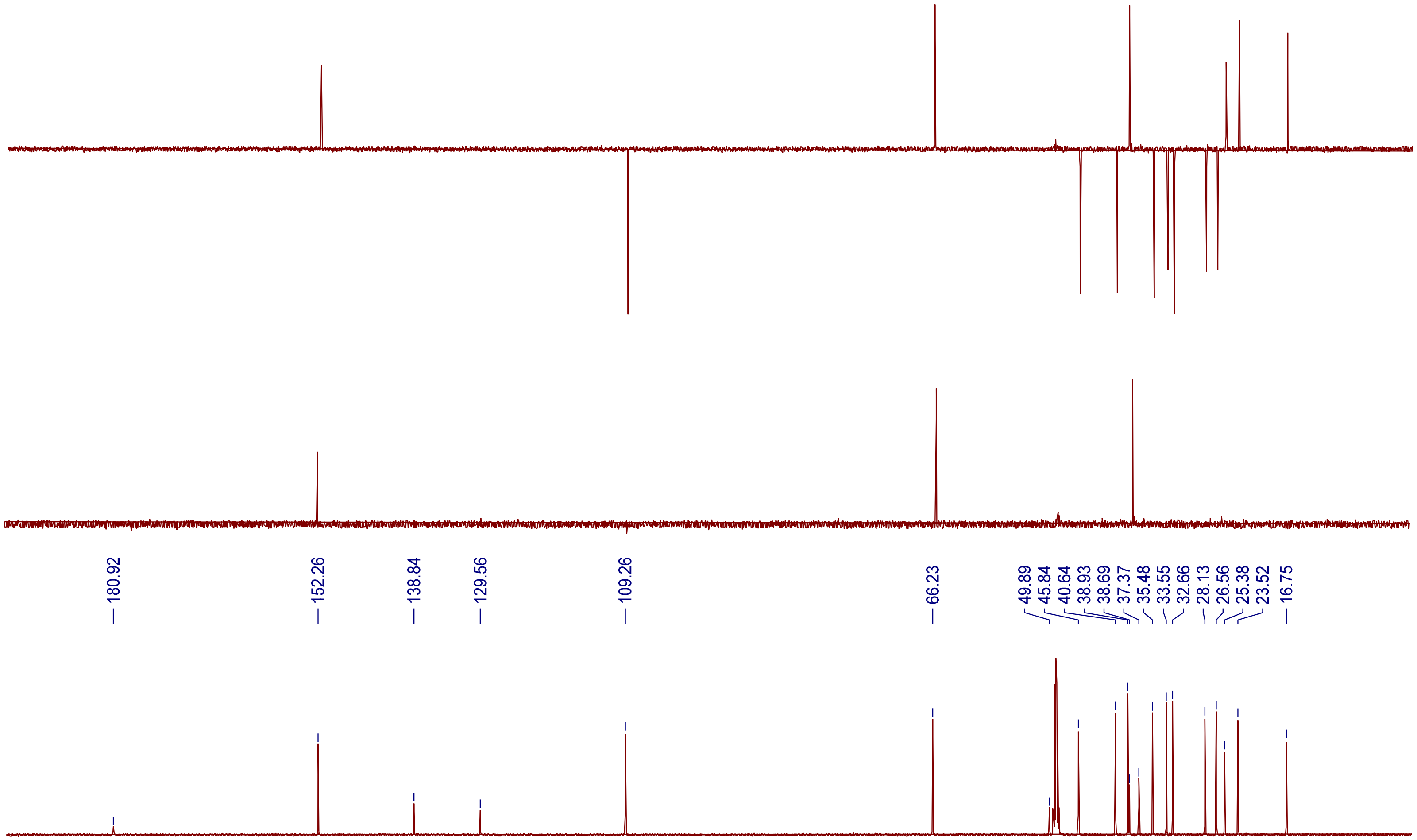

S10. HSQC spectrum (600 MHz, CD_3OD) of rosenolic acid (**2**)

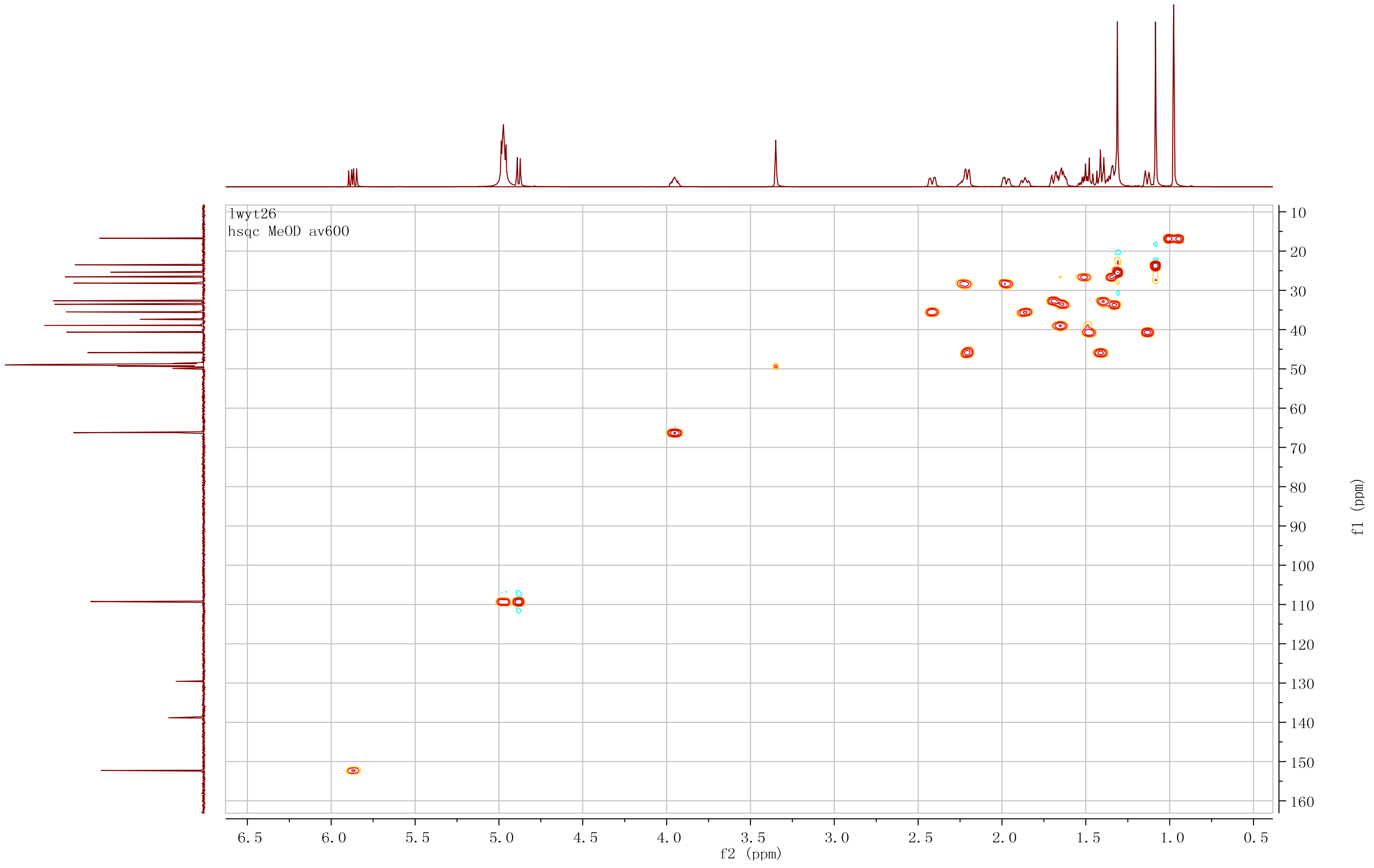

S11. HMBC spectrum (600 MHz, CD_3OD) of rosenolic acid (**2**)

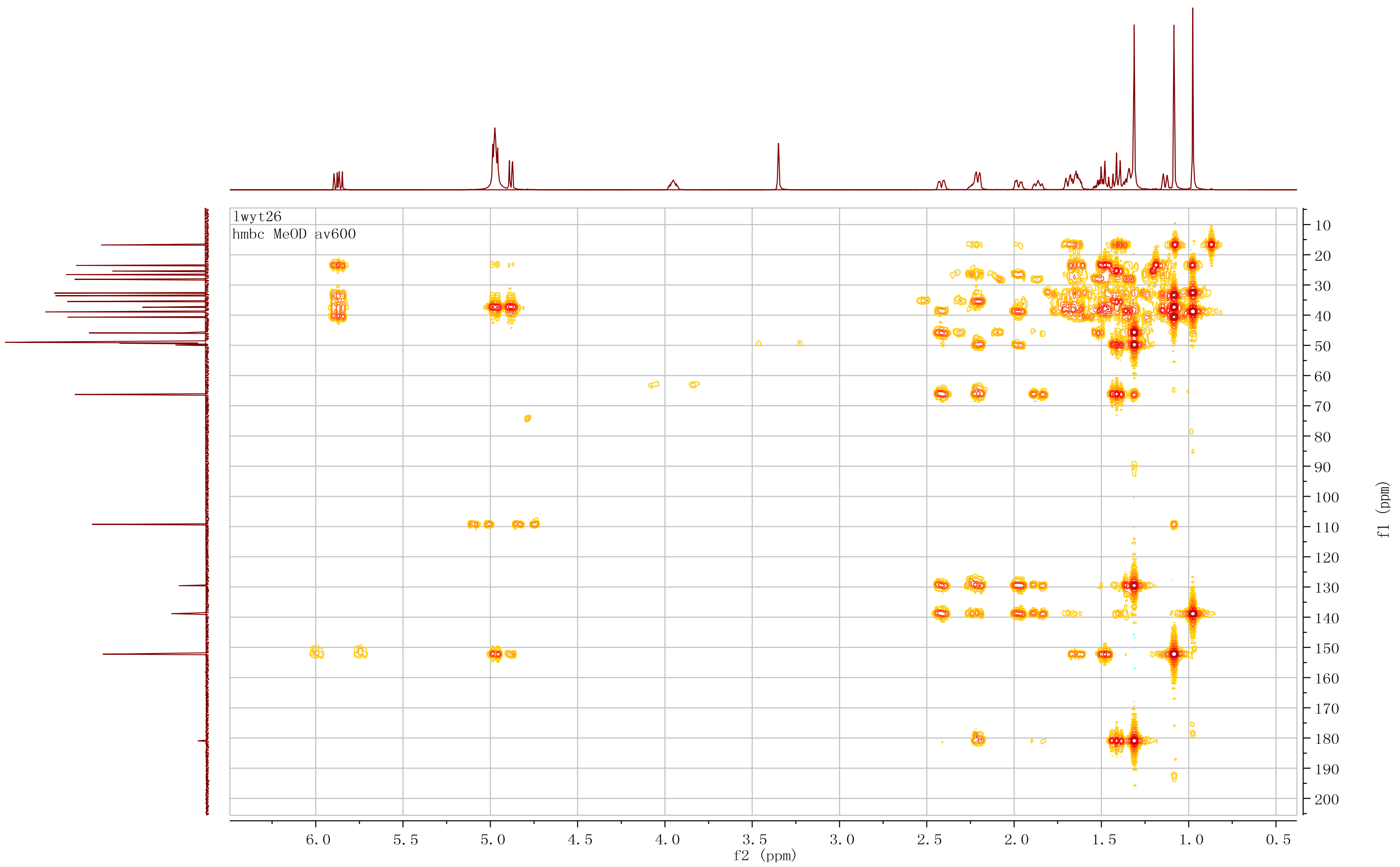

S12. COSY spectrum (600 MHz, CD_3OD) of rosenolic acid (**2**)

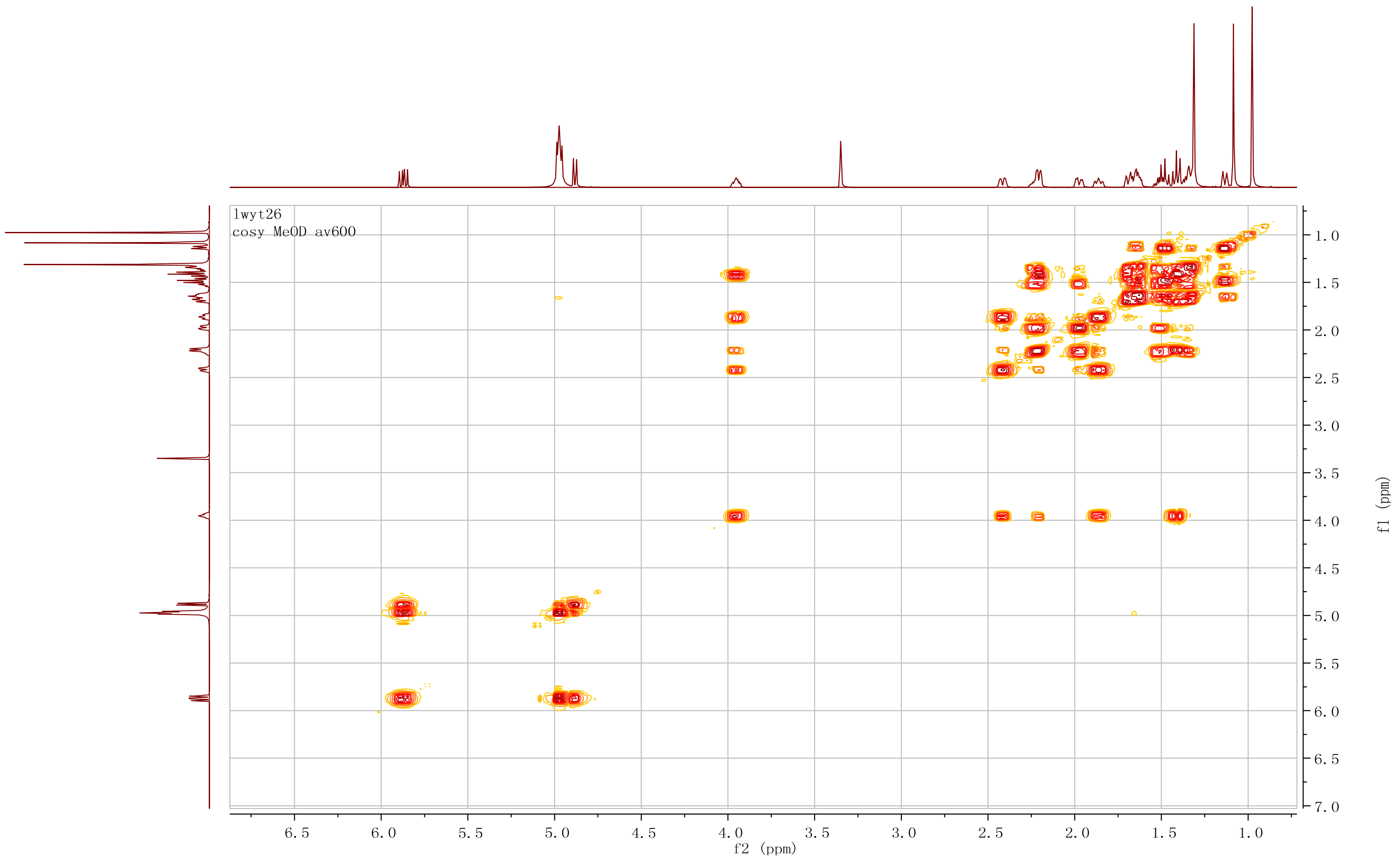

S13. ROSEY spectrum (600 MHz, CD_3OD) of rosenolic acid (**2**)

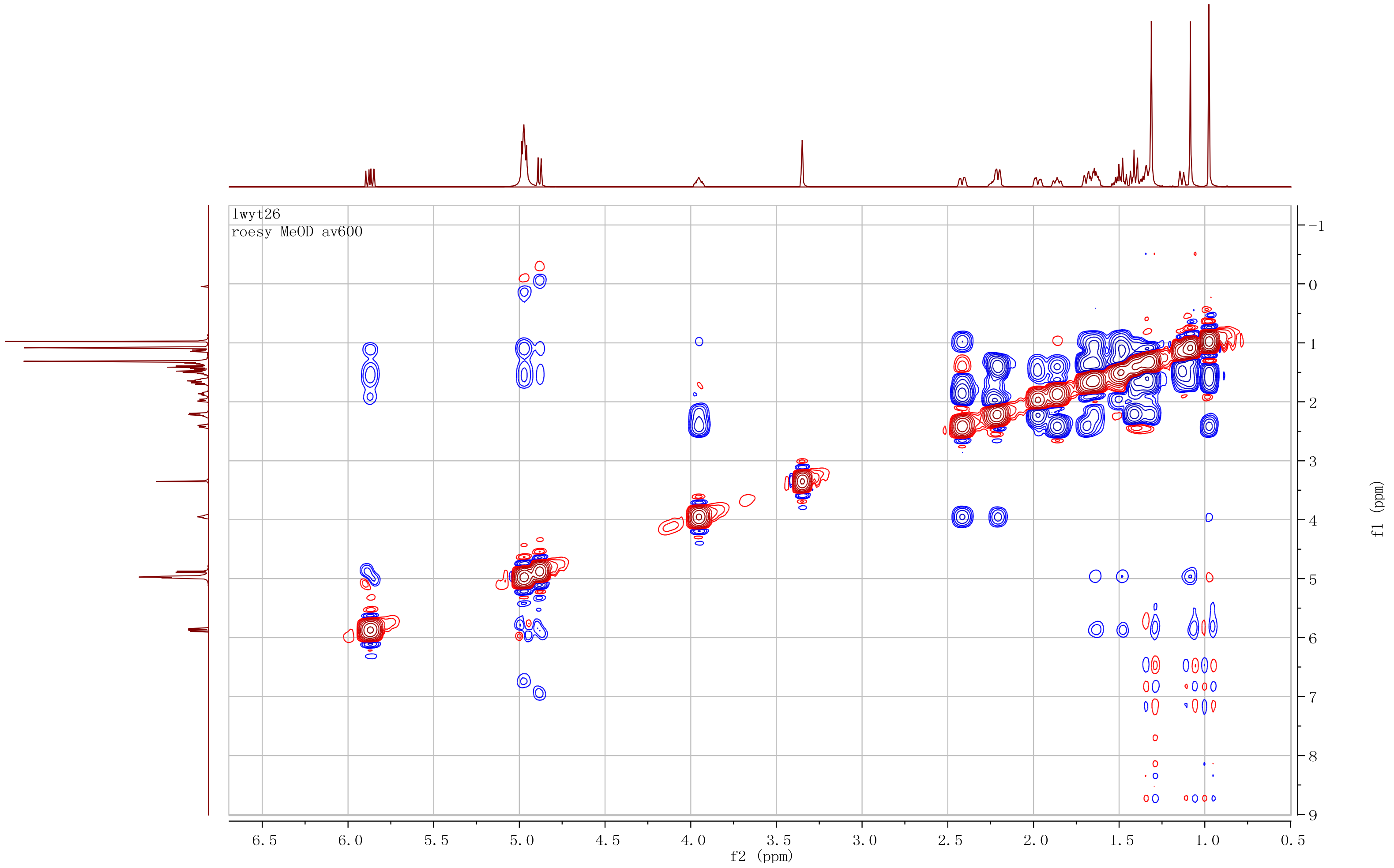

S14. HR-ESI-MS spectrum of rosenolic acid (**2**)

Qualitative Analysis Report

**Data Filename**	140804ESIA2.d	**Sample Name**	lwyt26
**Sample Type**	Sample	**Position**	
**Instrument Name**	Agilent G6230 TOF MS	**User Name**	KIB
**Acq Method**	ESI.m	**Acquired Time**	8/5/2014 9:52:18 AM
**IRM Calibration Status**	Success	**DA Method**	ESIN.m
**Comment**			

**Sample Group**		**Info.**
**Acquisition SW Version**	6200 series TOF/6500 series Q-TOF B.05.01 (B5125.1)	

User Spectra

Fragmentor Voltage	Collision Energy	Ionization Mode
200	0	ESI

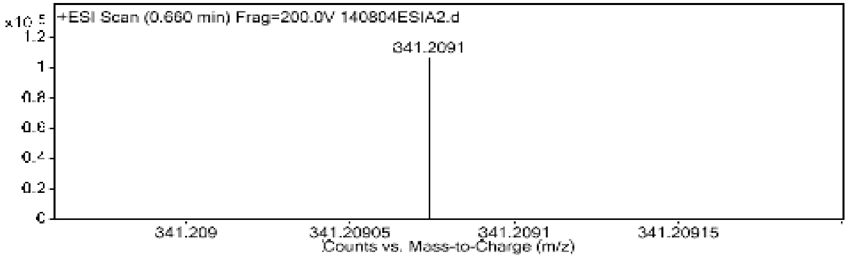

Peak List

m/z	z	Abund
274.2747	1	410710.31

Formula Calculator Element Limits

Element	Min	Max
C	0	200
H	0	400
O	1	10
Na	1	1

Formula Calculator Results

Formula	CalculatedMass	Mz	Diff.(mDa)	Diff. (ppm)	DBE
C20 H30 Na O3	341.2093	341.2091	0.2	0.6	5.5

--- End Of Report ---

S15. ^{1}H NMR spectrum (400 MHz, $CDCl_3$) of rosenolic dienol (**3**)

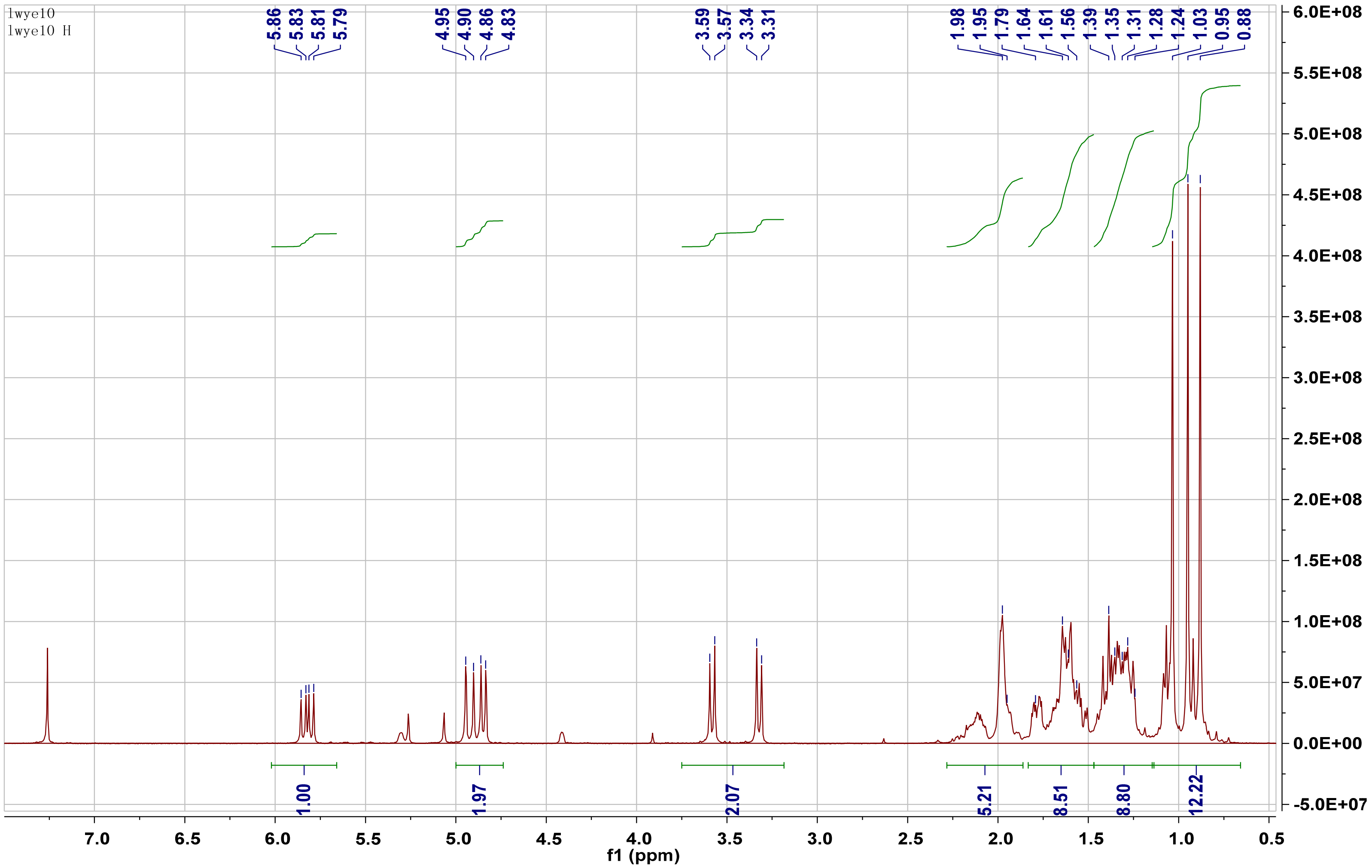

S16. ^{13}C NMR spectrum (100 MHz, $CDCl_3$) of rosenolic dienol (**3**)

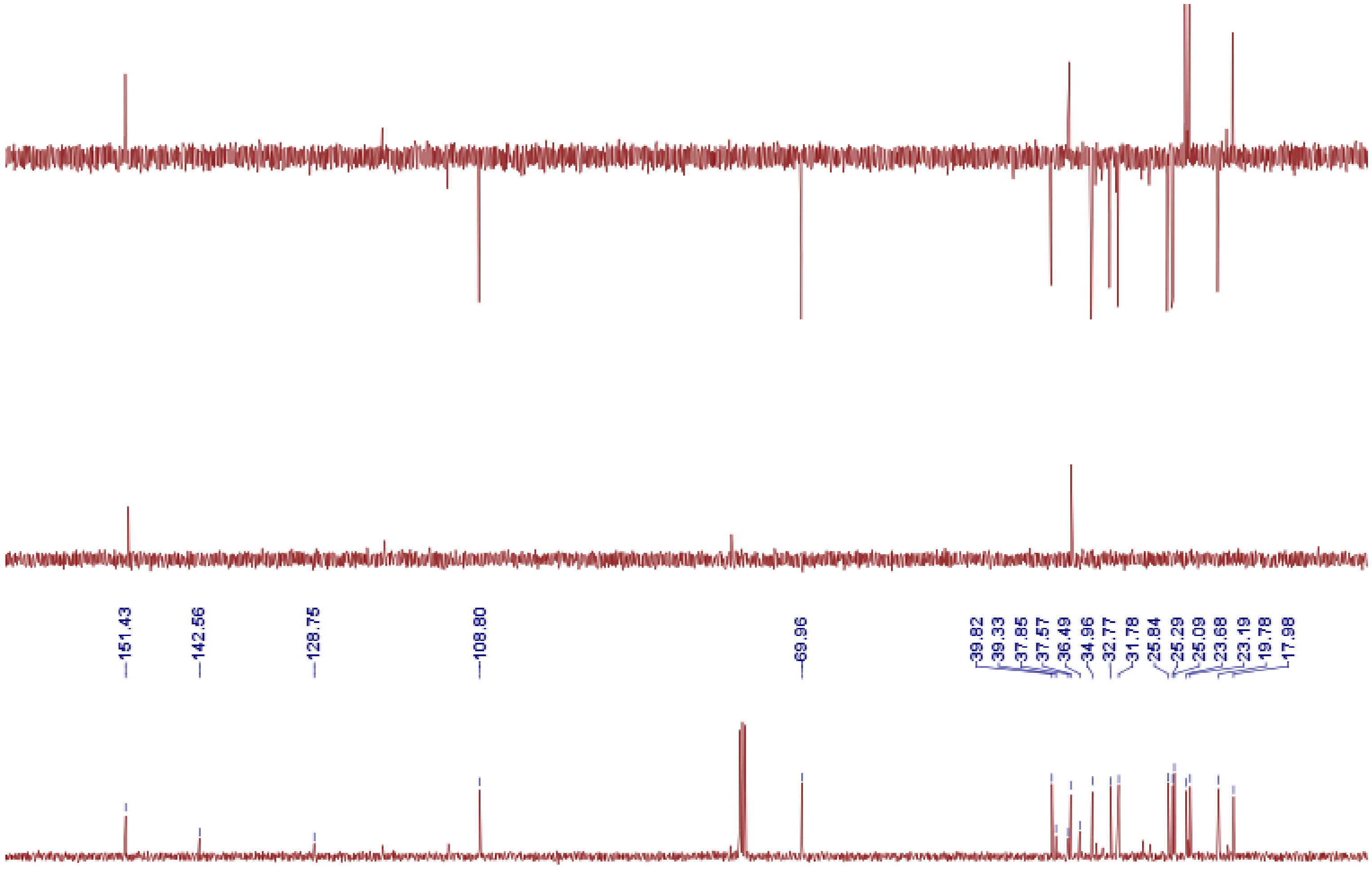

S17. HSQC spectrum (500 MHz, $CDCl_3$) of rosenolic dienol (**3**)

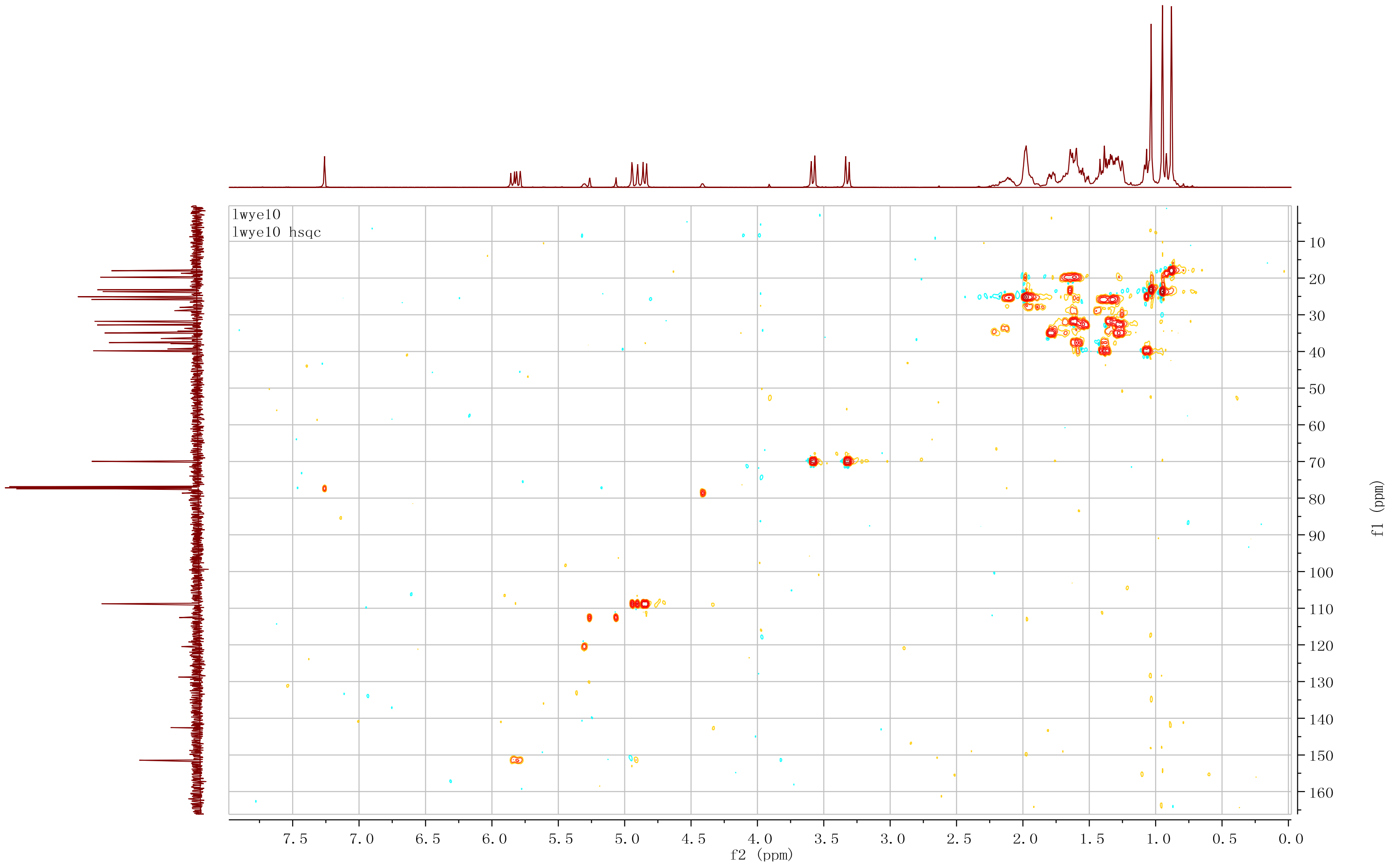

S18. HMBC spectrum (500 MHz, $CDCl_3$) of rosenolic dienol (**3**)

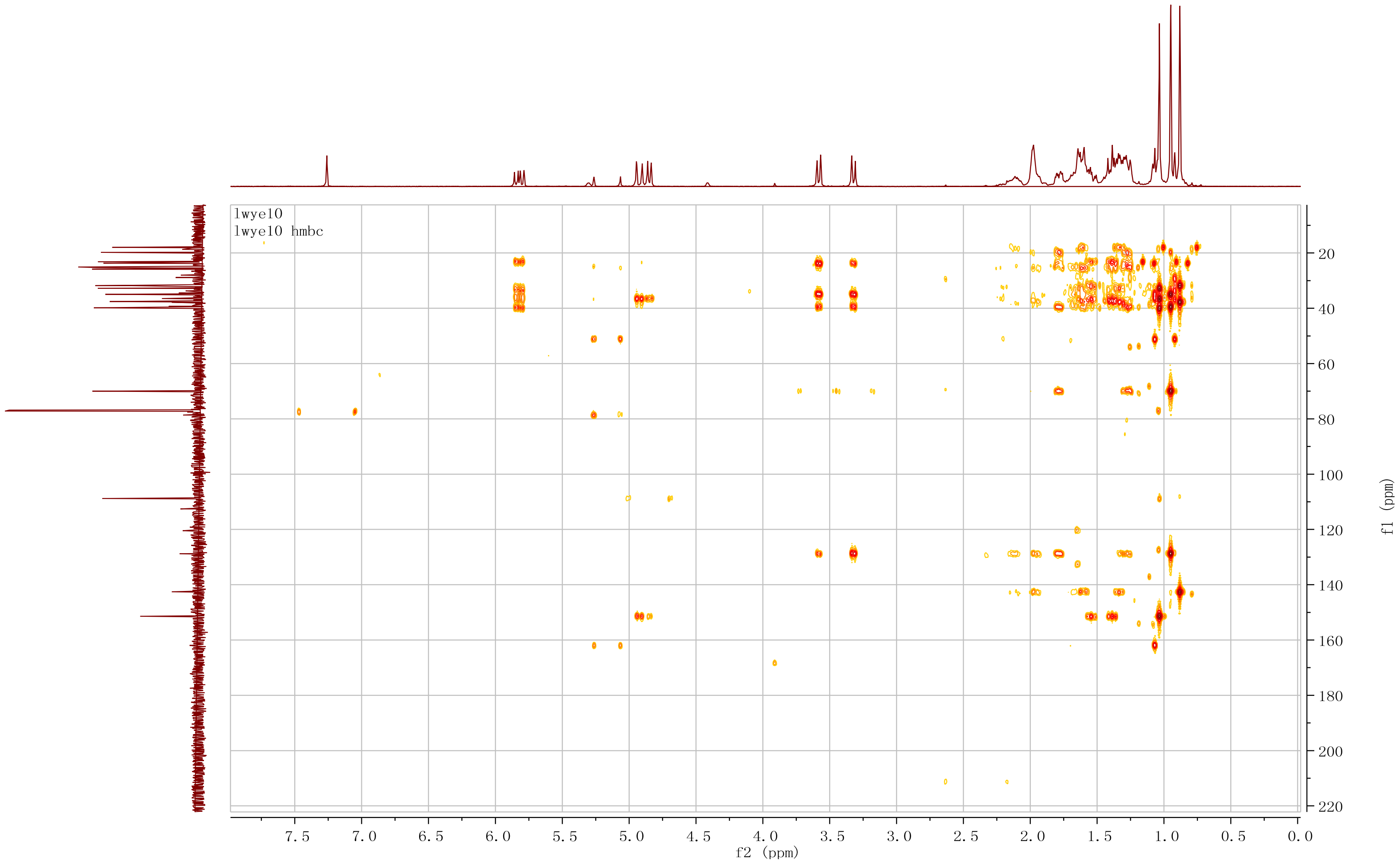

S19. COSY spectrum (500 MHz, $CDCl_3$) of rosenolic dienol (**3**)

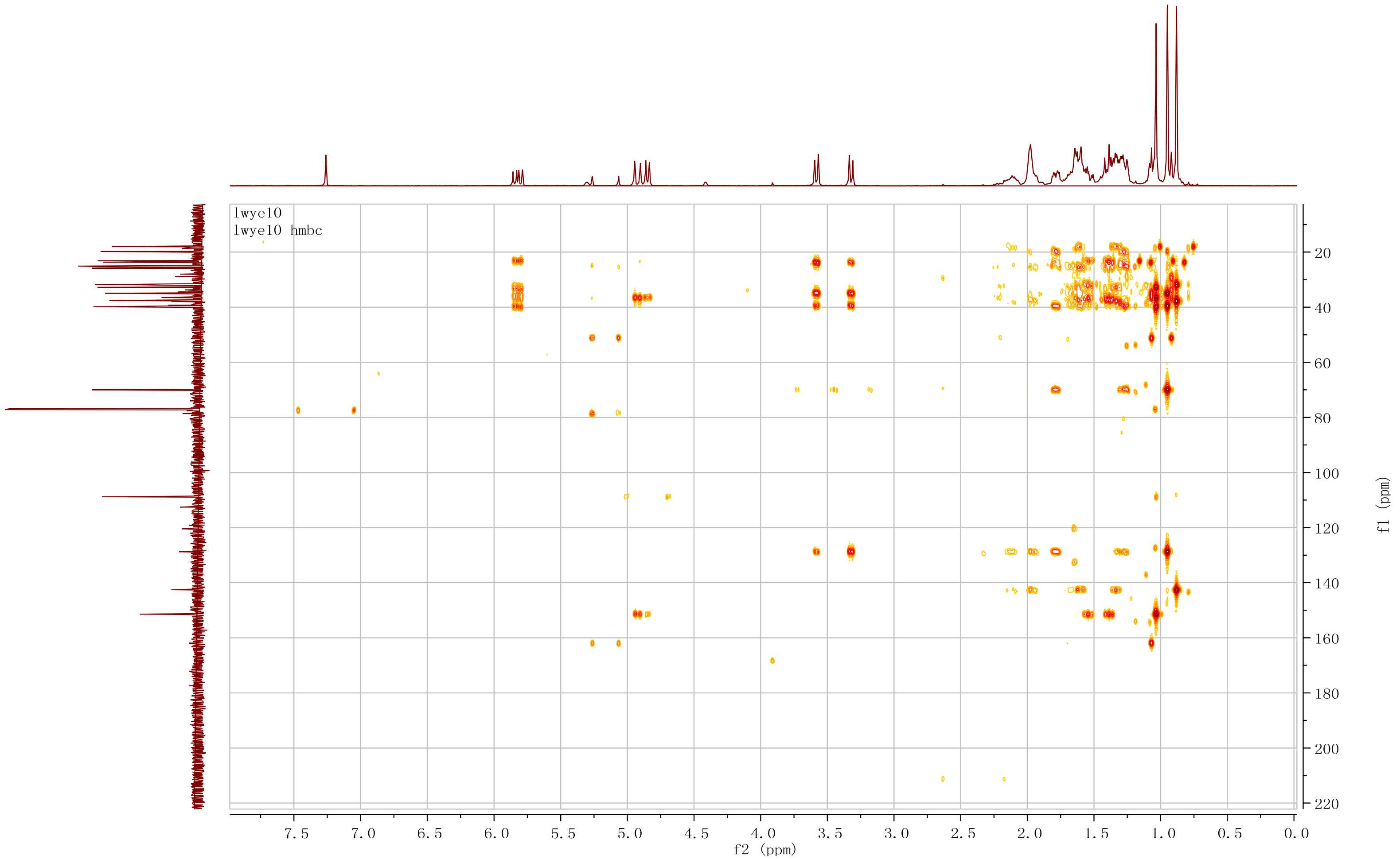

S20. ROSEY spectrum (500 MHz, $CDCl_3$) of rosenolic dienol (**3**)

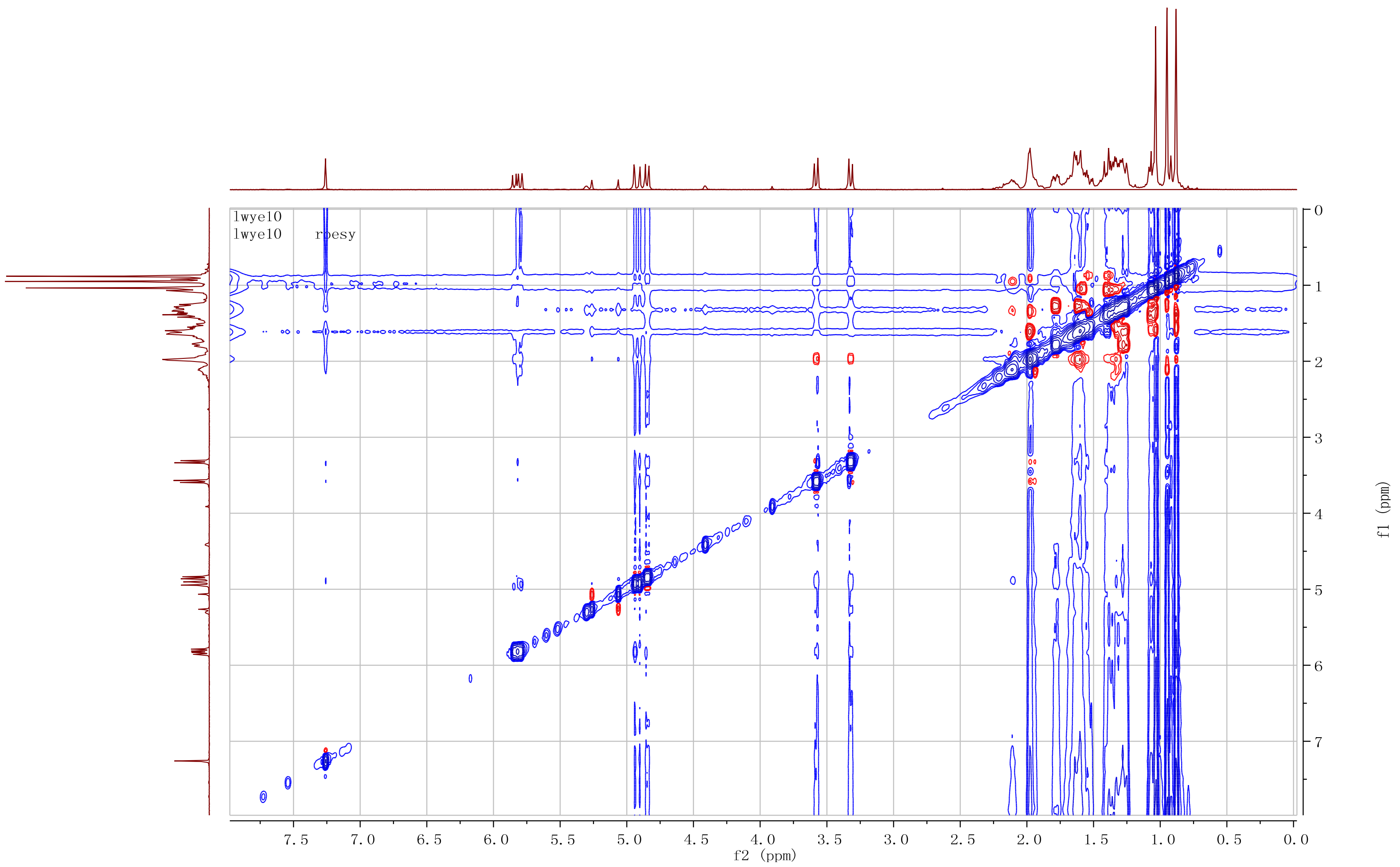

S21. HR-ESI-MS spectrum of rosenolic dienol (**3**)

Qualitative Analysis Report

**Data Filename**	140804ESIA1.d	**Sample Name**	lwyt29
**Sample Type**	Sample	**Position**	
**Instrument Name**	Agilent G6230 TOF MS	**User Name**	KIB
**Acq Method**	ESI.m	**Acquired Time**	8/5/2014 9:50:04 AM
**IRM Calibration Status**	Success	**DA Method**	ESIN.m
**Comment**			

Sample Group **Info.**
Acquisition SW 6200 series TOF/6500 series
Version Q-TOF B.05.01 (B5125.1)

User Spectra

Fragmentor Voltage	Collision Energy	Ionization Mode
200	0	ESI

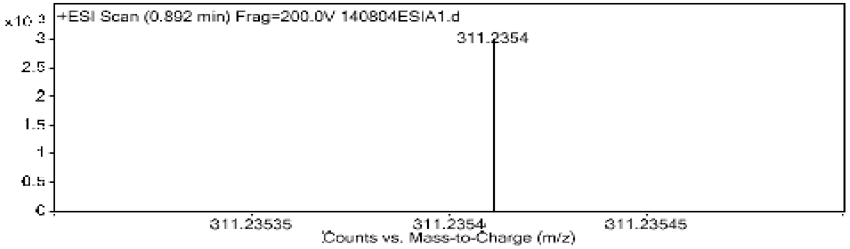

Peak List

m/z	z	Abund
274.2739	1	74430.61

Formula Calculator Element Limits

Element	Min	Max
C	0	200
H	0	400
O	1	3
Na	1	1

Formula Calculator Results

Formula	CalculatedMass	Mz	Diff.(mDa)	Diff. (ppm)	DBE
C20 H32 Na O	311.2351	311.2354	-0.3	1.0	4.5

--- End Of Report ---
